# Preclinical development of CD126 CAR-T cells with broad antitumor activity

**DOI:** 10.1038/s41408-020-00405-z

**Published:** 2021-01-04

**Authors:** Ameet K. Mishra, Iris Kemler, David Dingli

**Affiliations:** 1 Division of Hematology, 200 First Street SW, Mayo Clinic, Rochester, MN 55905 USA; 2 Department of Molecular Medicine, 200 First Street SW, Mayo Clinic, Rochester, MN 55905 USA

**Keywords:** Translational research, Immunotherapy

## Abstract

Chimeric antigen receptor T (CAR-T) cell therapy is a transformative approach to cancer eradication. CAR-T is expensive partly due to the restricted use of each CAR construct for specific tumors. Thus, a CAR construct with broad antitumor activity can be advantageous. We identified that CD126 is expressed by many hematologic and solid tumors, including multiple myeloma, lymphoma, acute myeloid leukemia, pancreatic and prostate adenocarcinoma, non-small cell lung cancer, and malignant melanoma among others. CAR-T cells targeting CD126 were generated and shown to kill many tumor cells in an antigen-specific manner and with efficiency directly proportional to CD126 expression. Soluble CD126 did not interfere with CAR-T cell killing. The CAR-T constructs bind murine CD126 but caused no weight loss or hepatotoxicity in mice. In multiple myeloma and prostate adenocarcinoma xenograft models, intravenously injected CD126 CAR-T cells infiltrated within, expanded, and killed tumor cells without toxicity. Binding of soluble interleukin-6 receptor (sIL-6R) by CAR-T cells could mitigate cytokine release syndrome. Murine SAA-3 levels were lower in mice injected with CD126 CAR-T compared to controls, suggesting that binding of sIL-6R by CAR-T cells could mitigate cytokine release syndrome. CD126 provides a novel therapeutic target for CAR-T cells for many tumors with a low risk of toxicity.

## Introduction

Chimeric antigen receptor (CAR) T cell therapy is emerging as a significant treatment modality for patients with a variety of relapsed/refractory malignancies. CAR-T cell therapies are often associated with impressive and rapid response rates, many times leading to a state where no malignant cells are detectable (minimal residual disease negativity)^1–6^. CAR-T cells targeting CD19 are now approved for both B cell non-Hodgkin lymphoma (Yescarta^TM^ and Kymriah^TM^) and also B cell acute lymphoblastic leukemia (Kymriah^TM^). CAR-T that targets other tumor-associated antigens such as B cell maturation antigen (BCMA) for multiple myeloma (MM)^7,8^, CD20 for lymphoma and chronic lymphocytic leukemia^9^ and CD123^10^ to target acute myeloid leukemia are showing promising results. Approaches to develop CAR-T cells targeting tumor-associated antigens that are overexpressed in a variety of “solid malignancies” such as CEA, MUC1, ERBB2, EGFR, and others are also developing at a rapid pace and will be in clinical trials soon^11,12^. Despite robust initial clinical responses, a significant number (30–60%) of patients experience relapse, and in a significant number (10–20%) of patients this is due to loss of antigen (CD19)^13^. To date, CAR-T cell products have been generated to treat a restricted number of tumors due to the limited expression of the target antigen (e.g., CD19 or BCMA). In principle, this approach can lead to higher costs of therapy since each individual target must be validated and is unlikely to be mass produced. Therefore, the identification and validation of a CAR-T cell target that is expressed by a wide variety of tumors would be highly desirable, since this will make such a construct applicable to many tumors and will likely reduce costs.

CD126 (interleukin-6 receptor (IL-6R)) and CD130 (IL-6ST) are the two known receptors responsible to transduce IL-6 signaling. CD126 is primarily expressed on B cells, epithelial cells, and hepatocytes^14^. CD130 is ubiquitously expressed but it does not directly bind IL-6, although its cytoplasmic domain is required for the initiation of IL-6 signaling^15^. IL-6 binds to CD126 forming an IL-6–CD126 complex that in association with CD130 initiates signaling. Cells that do not express CD126 can still respond to IL-6 since a soluble form of CD126 (sIL-6R) can bind IL-6 in solution to form an IL-6–sIL-6R complex, which can bind to CD130 expressed on other cells and induce a response (trans IL-6 signaling)^16,17^. A soluble form of CD130 (slL-6ST) acts as a decoy receptor^14,18^. In vitro, neutralizing antibodies against IL-6 increase apoptosis and decrease growth in primary myeloma cells as well as IL-6-dependent and autocrine human myeloma cell lines. In the following, we demonstrate the identification of CD126 as an antigen expressed by many tumors, including MM, lymphoma, and “solid tumors” that can be therapeutically targeted using CAR-T therapy.

## Methods

### Primary tumor and cancer cell line database

GEPIA2 (Gene Expression Profiling Interactive Analysis 2) database (Zhang lab, Peking University) was used to quantify overall survival in all cancer cases and acute myeloid leukemia (AML) based on CD126 expression^19^. Kaplan–Meier curve was used to calculate overall survival and RNA-sequencing was used to quantify CD126 expression. Cancer Cell Line Encyclopedia database was used to calculate CD126 expression across different cancer cell lines^20^.

### Primary tumor and cell lines

Studies involving the use of primary cells isolated from patients were conducted after approval from the Institutional Review Board at Mayo Clinic. Bone marrow specimens were collected from 24 patients with MM and the peripheral blood of five patients with acute lymphocytic leukemia (ALL). Normal plasma cells (CD138^+^CD27^+^) and B cells (CD19^+^B220^+^) were used as controls from seven healthy donors. The normal lung fibroblast cell line (MRC-5), human liver epithelial transformed cell line (THLE-3), and human retinal pigmented epithelial cell line (ARPE-19) were used as non-cancerous controls. Multiple cancer cell lines were used in this study: MM (RPMI-8226, U266B1, MM1.S, KMS-12-PE), AML (HL-60), non-Hodgkin lymphoma (SU-DHL-1, KARPAS 299), T cell leukemia (Jurkat, MOLT-4), chronic myeloid leukemia (K562), prostate cancer (DU145, LNCaP), malignant melanoma (624-mel), lung adenocarcinoma (A549), hepatocellular carcinoma (HepG2), and glioblastoma (U-251 MG). MM, AML, CML, lymphoma, and T cell leukemia cell lines were cultured in RPMI-1640 (Thermo Fisher). The MRC-5 and ARPE-19 and HEK293T cell lines were cultured in Dulbecco’s modified Eagle’s medium (Thermo Fisher). THLE-3 cells were cultured in bronchial epithelial cell growth medium (Lonza). All cell culture media were supplemented with 10% fetal bovine serum (Thermo Fisher) together with penicillin (100 U/ml) and streptomycin (100 μg/ml). Prior to all the in vivo experiments, all cell lines were tested at IDEXX Bioanalytics for the presence of mycoplasma or murine viruses.

### CAR construction and lentivirus preparation

Two scFv-targeting (VQ8F11 and rhMP-1) CD126 were used to construct third-generation CAR (Creative Biolabs). The lentiviral vector construct contains the EF-1α promoter, CD28 transmembrane domain, and 4-1BB co-stimulatory region (Fig. 2a). VQ8F11 has a stronger binding affinity to CD126 compared to rhMP-1. rhMP-1 is biosimilar to tocilizumab and can bind to soluble CD126. Lentivirus particles were generated in HEK293T cells by transduction with a three-plasmid system coding for the lentivector genome, pMD2.G (Addgene), and psPAX2 (Addgene) using HD Fugene (Promega). Lentiviral vectors were harvested at 48 and 72 h after transduction, concentrated by ultracentrifugation and titrated on HEK293T for further downstream use. All vector preparations were frozen at −80 °C until they were required.

### Human T cell transduction

Primary human T cells were isolated from leukoreduction cones^21^ from healthy donors after approval from the Institutional Review Board at Mayo Clinic. T cells were negatively selected using magnetic beads (BioLegend), stimulated with CD3/CD28 Dynabeads (Thermo Fisher) at a 3:1 (beads to cell) ratio for 5–6 days and grown in the presence of IL-7 (5 ng/ml) and IL-15 (5 ng/ml). T cells were transduced on retronectin (Takara)-coated plates with concentrated lentivirus vector on days 2 and 3. Transduction efficiency was determined on days 7–10 with a flow cytometry using a biotinylated recombinant human CD126 and mCherry reporter genes. T cells were expanded for 2 weeks before downstream experiments.

### Bioluminescence-based cytotoxicity assay

Luciferase-expressing cancer cell lines were established using p-Ultra-Chili-Luc (Addgene) lentiviral vector. Mock T cells or CAR-T cells were seeded in a 96-well plate with cancer cells at different effector-to-target ratios. d-Luciferin potassium salt (75 μg/ml) was added to each well. Wells containing tumor cells without T cells served as controls for spontaneous cell death, and for the maximal killing control, wells contained cancer cells with lysis buffer. Bioluminescence was measured after 16 h with a luminometer (Infinite M200 Pro, Tecan) as relative light units (RLUs). Specific killing was calculated using the following equation: percentage specific killing = 100 × (spontaneous cell death RLU − sample RLU)/(spontaneous death RLU − maximum killing RLU).

### Calcein-acetyoxymethyl (Calcein-AM) cytotoxicity assay

Cytotoxicity for primary tumor cells was performed with the Calcein-AM cytotoxicity assay as per the manufacturer’s recommendations. The cells were labelled with Calcein-AM and subsequently cultured either with mock T cells or CAR-T at different effector-to-target ratios. For primary MM, an effector-to-target ratio of 10:1 only was studied due to insufficient number of cells. Plates were read after 16 h of incubation with a fluorescence plate reader. Similar controls and the same equation as in the bioluminescence-based cytotoxicity assay were used.

### Flow cytometry

Single-cell suspensions were stained for cell surface expression using one of the following conjugated antibodies: anti-CD126 (UV4), anti-CD3 (HIT3a), anti-CD138 (DL-101), anti-CD27 (M-T271), anti-CD19 (4G7), and anti-B220 (RA3-6B2). CAR-T binding affinity with recombinant human CD126 and murine CD126 were quantified using biotinylated protein and anti-biotin-conjugated secondary antibodies. Data were acquired on LSR Fortessa (Becton Dickinson) and ZE5 (Bio-Rad), and analyzed using FlowJo v.10.7 (FlowJo, BD Biosciences).

### CD126-targeted CAR-T toxicity on autologous peripheral blood mononuclear cell (PBMC)

PBMCs were isolated from leukocyte reduction cones and labelled with carboxyfluorescein succinimidyl ester. Autologous mock T cell and CD126-targeted CAR-T cells were labelled with cell trace violet. Donor-matched PBMCs were co-cultured with mock T cells or CD126-targeted CAR-T cells at a fixed effector-to-target ratio for 24 h. Cells were stained with anti-CD19, anti-CD3, anti-CD14, and anti-CD56 antibodies. RPMI-8226 cells were co-cultured with mock or CD126 targeting CAR-T cells as a positive control in the assays. Data were acquired on LSR Fortessa and analyzed with FlowJo v.10.7.

### Liver function testing and cytokine profiling

Blood was collected from mice at the end of the experiment by performing cardiac puncture and plasma was collected by centrifugation. Aspartate aminotransferase was quantified with an enzyme-linked immunosorbent assay according to the manufacturer’s protocol (aspartate aminotransferase, Sigma-Aldrich). Mouse SAA-3 (Millipore Sigma), human interferon-γ (Invitrogen), and sIL-6Rα (Invitrogen) were quantified in plasma or conditioned media using established enzyme-linked immunosorbent assays.

### Murine experiments

All murine experiments were conducted in compliance with the Institutional Animal Care and Use Committee approval at Mayo Clinic Rochester. Six- to 12-week-old NSG (NOD.Cg-PrkdcscidIl2rgtm1Wjl/SzJ) mice (The Jackson Laboratory) were used. In order to test the safety of the generated CAR-T cells, the mice were injected either with 1 × 10^7^ mock T cells or CAR-T cells intravenously via the tail vein. Their baseline and subsequent body weight was monitored daily for 2 weeks. Subsequently, the mice were euthanized by carbon dioxide inhalation and the livers were excised, weighed, and liver function tests was performed from blood drawn as a terminal bleed.

In order to determine the in vivo activity of the CD126 targeting CAR-T cells, ten million RPMI-8226 or DU145 (ultrachilli-luciferase) cells were injected subcutaneously in both flanks. After 4 days, 1 × 10^7^ mock T cells or CAR-T cells were administrated by tail vein injection once. Bioluminescence imaging was performed with Xenogen IVIS Imaging System (Xenogen) after the intraperitoneal injection of d-luciferin (15 mg/ml). The tumor burden was assessed using Living Image software (Xenogen). At the end of the experiment, the mice were euthanized, the tumors were identified, and excised. Some of the tumors was lysed and suspended in phosphate-buffered saline for flow cytometry.

### Statistical analysis

Statistical analysis was performed using Prism 8 (GraphPad Prism software). Differences between groups were tested using the Mann–Whitney test and analysis of variance was used to compare more than two groups. A *P* < 0.05 was considered statistically significant.

## Results

### CD126 is ubiquitously expressed on tumor cells and is a prognostic marker

**Fig. 1:**
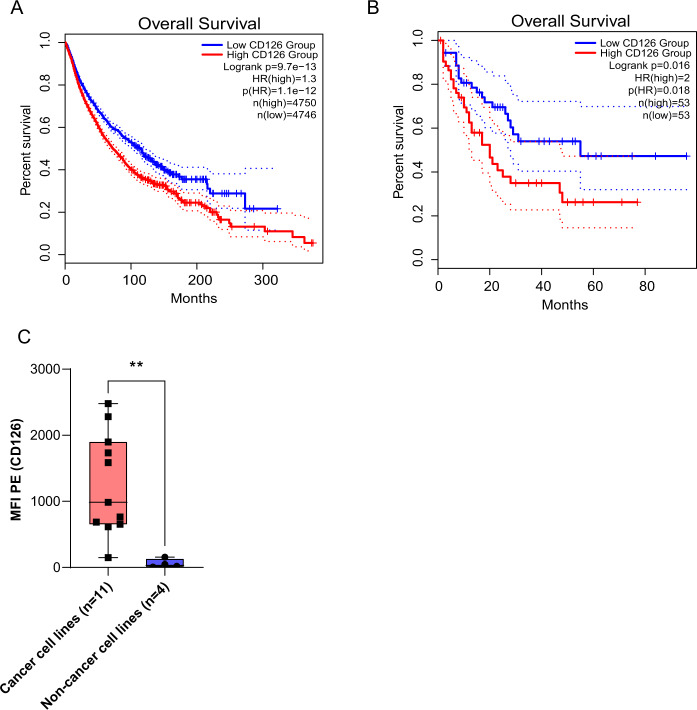
CD126 is ubiquitously expressed on tumor cells and is a prognostic marker. **a** Overall survival in various types of cancer (*n* = 9496) based on high and low CD126 expression. Median CD126 expression (RNA-sequencing) was used to differentiate CD126 expression. **b** Overall survival in AML (*n* = 106) based on high and low CD126 expression. **c** MFI CD126 expression in cancer cell lines (*n* = 11) compared to non-cancer cell lines (*n* = 4). Mann–Whitney *U* test. **P < 0.01. Each black square is an individual data point..

In our search of a pan-cancer surface target for immunotherapy, we identified CD126 as a potential candidate. It is ubiquitously expressed on cancer cells but has limited expression on many essential cells. We analyzed RNA-sequencing data from the Cancer Cell Line Encyclopedia database^20^. IL-6R messenger RNA expression was significantly higher in human myeloma cell lines (*p* < 0.001) compared to other hematopoietic and lymphatic tumors as well as other cancers (Supplementary Fig. 1A). We used GEPIA2, and evaluated the expression of CD126 in a wide variety of tumors. We found that many tumors overexpress this antigen even if they are not thought to depend or respond to IL-6. The dataset (*n* = 9496) was dichotomized into two cohorts with patients’ tumors having high or low CD126 expression using the median as a divider. Subsequently, we evaluated the impact of CD126 expression on overall survival. Cases with high CD126 expression had a significantly lower overall survival (Fig. 1a) with the difference being quite dramatic in AML (Fig. 1b). We quantified cell surface CD126 expression in primary MM cells (*n* = 24) and ALL (*n* = 5). Compared to normal plasma cells and B cells (*n* = 7), we observed a trend towards higher CD126 expression on primary myeloma and ALL cells, but this did not reach statistical significance (Supplementary Fig. 1B, C). In the same database, CD126 expression was significantly higher (6-fold) on AML cells (*n* = 173) compared to normal B cells (*n* = 70) (Supplementary Fig. 1D). We also found that CD126 expression was significantly higher on cancer cell lines (RPMI, KMS12PE, U266, ANBL6, OPMI, OPM2, MM1S, HML60, JJN3, U-251, DU145) (*n* = 11) compared to transformed normal cell lines (*n* = 4) (293T, THELE-3, MRC-5, ARPE-19) (Fig. 1c).

### Development of a CD126-targeted CAR

**Fig. 2:**
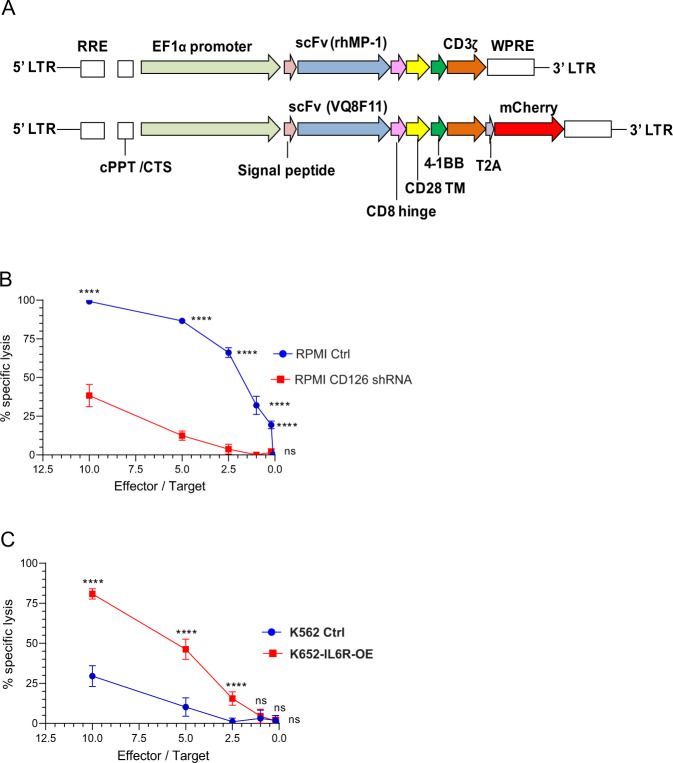
Development of a CD126-targeted CAR. **a** Schematic representation of CAR constructs used in this study. **b** CAR-T cell-mediated killing of RPMI and a derivative population with suppressed CD126 expression using shRNA (RPMI-CD126 shRNA) both expressing luciferase incubated with CAR-T cells at different effector-to-target ratios (*n* = 12). **c** Cell killing of K562 and K562 that express CD126 (K562 CD126 OE) and luciferase with CAR-T cells at different effector-to-target ratios (*n* = 12). Data are mean ± standard deviation. Two-way ANOVA and multiple comparison test. n.s. Not significant. ****P < 0.0001.

We selected two scFv (VQ8F11 and rhMP-1) that target CD126 to construct third-generation CAR (Fig. 2a) using lentiviral vectors. The scFv rhMP-1 targets the same epitope as tocilizumab and has a lower binding affinity than the VQ8F11 scFv. mCherry was used as a reporter gene in the VQ8F11 CAR. Transduction efficiency on primary T cells ranged from 30 to 70%, and was typically ~50% (Supplementary Fig. 2A, B). We did not observe a significant difference in tumor killing (in vitro) between the two CAR-T constructs (Supplementary Fig. 2E–G). As a result, all subsequent experiments were performed with the rhMP-1 CAR-T cells.

In order to determine the specificity of CD126 CAR-T cells, we used CD126 knockdown RPMI cells and CD126-overexpressing K562 using lentiviral short hairpin RNA (shRNA) and CD126 complementary DNA open-reading frame vectors, respectively, as described before^22^ (Supplementary Fig. 2C, D). To determine the specificity of the CAR construct, we performed a bioluminescence-based cytotoxicity assay at effector/target ratios of 10, 5, 2.5, and 1. RPMI cells expressing a high level of CD126 showed significant specific lysis compared to the CD126 low expressing RPMI subclone (Fig. 2b). Conversely, when K562 was engineered to express CD126, specific lysis significantly increased at effector-to-target ratios of 10, 5, and 2.5 (Fig. 2c).

### CD126-targeted CAR-T cells demonstrate potent in vitro activity against multiple cancer cell lines

**Fig. 3:**
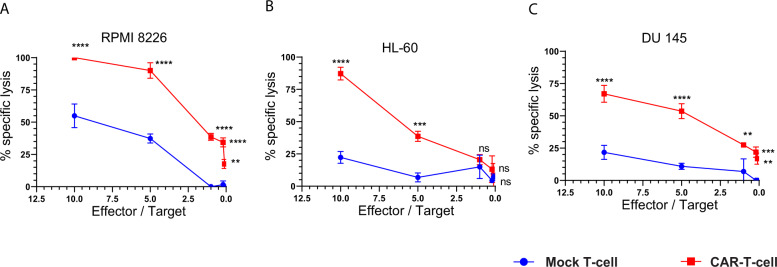
CD126-targeted CAR-T cells demonstrated potent in vitro activity against multiple tumor cell lines. Bioluminescence cell killing was performed in ultrachilli-luciferase-expressing cancer cell lines, **a** RPMI, **b** HL-60, and **c** DU145 when co-cultured with either CAR-T or mock T cell at effector-to-target ratios of 10, 5, 1, 0.2, and 0.1 (*n* = 12). Percent specific lysis was quantified after 16 hours of incubation. Two-way ANOVA and multiple comparison test. n.s. Not significant. **P < 0.01, ***P < 0.001, and ****P < 0.0001.

We tested the in vitro cell killing activity of primary human T cells and CD126-targeted CAR-T cells after co-culture with 15 different tumor cell lines, including MM (RPMI-8226, U266B1, MM1.S, KMS-12-PE), AML (HL-60), lymphoma (SU-DHL-1, KARPAS 299), T cell leukemia (Jurkat, MOLT-4), CML (K562), prostate cancer (DU145, LNCaP), malignant melanoma (624-mel), lung adenocarcinoma (A549), hepatocellular carcinoma (HepG2), and glioblastoma (U-251 MG). In all cell lines except U-251, we observed significantly higher specific lysis at effector-to-target ratios of 10 and 5 with the CD126-targeted CAR-T cells compared to mock T cells from the same donor (Fig. 3 and Supplementary Fig. 3). We also observed the specific killing of RPMI-8226, DU145, and LNCaP, at lower effector-to-target ratios (0.2 and 0.1).

### CD126-targeted CAR-T cells demonstrate potent in vitro activity against primary MM and ALL cells

**Fig. 4:**
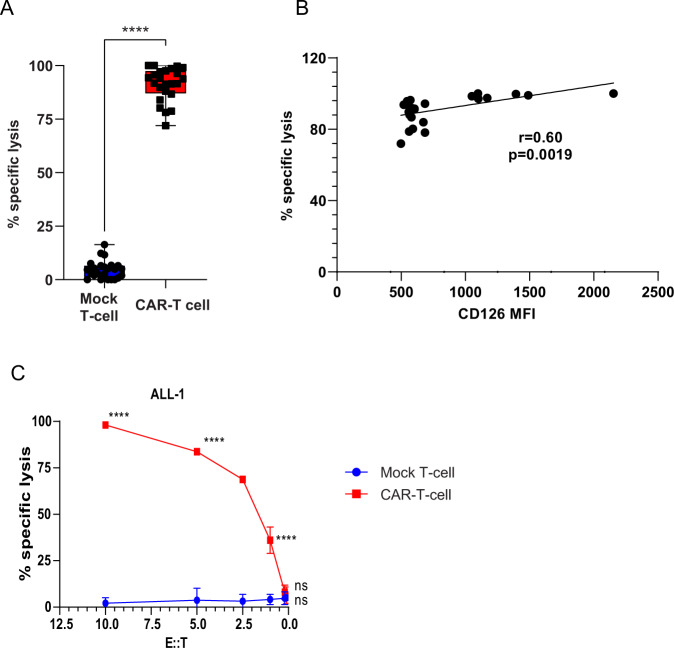
CD126-targeted CAR-T cells demonstrated potent in vitro activity against primary multiple myeloma and ALL cells. **a** Cell killing was measured in calcein-AM-labelled MM cells when co-cultured with either CAR-T or mock T cells at effector-to-target ratio of 10 after 16 h of incubation (*n* = 24 MM, 6 for each MM samples). **b** Lysis of target cells correlated with the level of CD126 expression. Spearman correlation coefficient was calculated between percentage specific lysis and CD126 MFI expression on primary myeloma cells (*n* = 24). **c** Cell killing of primary ALL cells was measured by calcein-AM-labelled cells when co-cultured with either CAR-T or mock T cell at effector-to-target ratio of 10, 5, 2.5, 1, 0.2, and 0.1 after 16 h of incubation (*n* = 10). Two-way ANOVA and multiple comparison test. n.s. Non-significant. ****P < 0.0001. Each black square or circle represents a single data point from the experiment.

To further confirm the in vitro data, we co-cultured primary MM cells and ALL cells after calcein-AM labelling, with either mock T cells or CD126-targeted CAR-T cells at various effector-to-target ratios. In MM (*n* = 22), we only used effector-to-target ratio of 10 due to insufficient number of cells. CAR-T cells significantly killed tumor cells (75–100%) compared to mock T cells (0–20%) (Fig. 4a). Specific lysis positively correlated with CD126 expression (*r* = 0.6, *p* = 0.0019) (Fig. 4b). In ALL samples (*n* = 5), we observed significantly higher specific lysis at effector-to-target ratio of 10, 5, 2.5, and 1 with CAR-T cells compared to mock T cells (Fig. 4c and Supplementary Fig. 4).

### CD126-targeted CAR-T cells bind to sIL-6R and do not cause toxicity in mice

sIL-6R is present in normal human plasma. Many cell lines that express high levels of IL-6R on their surface also secrete sIL-6R (Supplementary Fig. 5A). In order to determine whether CD126-targeted CAR-T cells bind to sIL-6R, we incubated mock T cells or CAR-T cells with conditioned media from RPMI-8226, HL-60, DU145, and K562 cells for 12 h and quantified unbound sIL-6R in the culture media. CAR-T cells significantly reduced sIL-6R compared to mock T cells. Normal physiological levels of sIL-6R are in the range of 20–75 ng/ml and may in principle interfere with CAR-T-mediated tumor killing. In order to test this hypothesis, we performed bioluminescent cytotoxicity assays of CAR-T cells against specific target cells in the presence and absence of recombinant human sIL-6R (50 ng/ml). We observed no difference in the efficacy of CAR-T cells either in the presence or absence of sIL-6R at effector-to-target ratios of 10, 5, 2.5, and 1 (Supplementary Fig. 5B).

Given that IL-6R is expressed on liver cells, we utilized a murine model to determine the potential hepatotoxicity of CD126-targeted CAR-T cells. Our CD126-targeted CAR binds with recombinant murine IL-6R (Supplementary Fig. 5C). Therefore, off-target effects and potential hepatotoxicity of CAR-T cells can be determined in the murine model. NSG mice were injected intravenously either with human T cells or CD126-targeted CAR-T cells and monitored for 2 weeks. There was no significant difference in body weight (Supplementary Fig. 5D), liver weight (Supplementary Fig. 5E), aspartate aminotransferase (Supplementary Fig. 5F) at the end of the 2-week observation period.

### CD126-targeted CAR-T cells do not eliminate autologous PBMCs, B cells, T cells, monocytes, or natural killer (NK) cells

In order to determine whether CD126-targeted CAR-T cells kill autologous PBMCs or other immune cells, we isolated PBMCs from three donors and generated mock and CAR-T cells targeting CD126. We incubated carboxyfluorescein succinimidyl ester-labelled PBMCs either with autologous mock T cells or CD126-targeted CAR-T cells for 24 h. Mock T cells and CAR-T cells were labelled with cell trace violet to distinguish them from the autologous PBMCs. In parallel, RPMI cells were co-cultured with CAR-T cells to serve as positive controls: the RPMI cells were killed by the CD126-targeted CAR-T cells compared to when co-cultured with mock T cells (Supplementary Fig. 6E). In contrast, CD126-directed CAR-T cells did not kill total PBMCs, B cells, T cells, monocytes, and NK cells (Supplementary Fig. 6E). Therefore, we can conclude that CD126-targeted CAR-T cells have no detrimental effects on autologous B or T cells, monocytes, and NK cells or other cellular components present in PBMCs.

### CD126-targeted CAR-T cells induce potent tumor regression in murine models of MM and prostate cancer

**Fig. 5:**
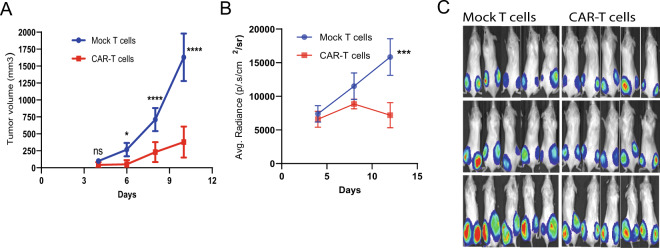
CD126-targeted CAR-T cells demonstrated potent antitumor activity in murine models of multiple myeloma and prostate cancer. NSG mice were injected subcutaneously with RPMI-ultrachilli-luciferase cells and treated with a single dose of CAR-T cells or mock T cells. **a** Tumor volume was measured with calipers (*n* = 10 each group). **b** Tumor burden (d-luciferin BLI of RPMI-ultrachilli-luciferase) mice were quantified on days 4, 8, and 12. **c** Representative images from five mice are shown from RPMI xenograft model. Mann–Whitney *U* non-parametric test. Two-way ANOVA and multiple comparison test. n.s. Not significant. *P < 0.05, ***P < 0.001, and ****P < 0.0001.

**Fig. 6:**
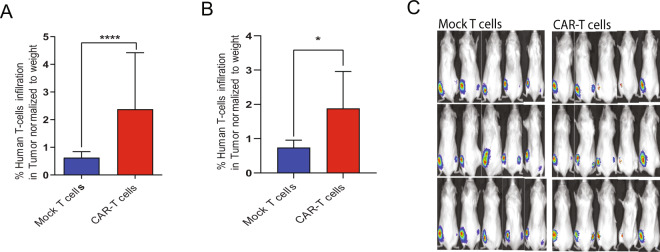
CD126-targeted CAR-T cells demonstrated potent antitumor activity in murine models of multiple myeloma and prostate cancer. **a** Tumor-infiltrating human T cells were quantified by anti-human CD3 antibody using flow cytometry in RPMI tumors explanted from mice that either received mock T cells (*n* = 8) or CAR-T cells (*n* = 10). **b** Tumor-infiltrating human T cells was quantified by anti-human CD3 antibody using flow cytometry of DU145-explanted tumors from mice that either received mock T cells (*n* = 5) or CAR-T cells (*n* = 4). **c** Representative images from five mice are shown from DU145 xenograft model. Mann–Whitney *U* non-parametric test. *P < 0.05 and ****P < 0.0001.

To evaluate the in vivo activity of CD126-targeted CAR-T therapy, we used MM (RPMI-8226) and prostate cancer (DU145) xenograft models. NSG mice were injected with ten million ultrachilli-luciferase RPMI cells or DU145 cells subcutaneously in both flanks. Four days after tumor implantation, the mice received a single dose of either 10^7^ T cells or CAR-T cells derived from the same donor via tail vein injection. In the aggressive RPMI model, we observed a significant reduction in the rate of tumor growth (Fig. 5a) and regression based on bioluminescence imaging (Fig. 5b) within 1 week of CAR-T therapy. T cell infiltration in the tumor was significantly higher in CD126-targeted CAR-T-injected group (Fig. 6a). In the slower-growing prostate cancer DU145 model, a single dose of CAR-T therapy reduced tumor burden compared to the mock T cell-injected group (Fig. 6c and Supplementary Fig. 6A). Human interferon-γ level was significantly higher, while SAA-3 was significantly lower in plasma of mice injected with CAR-T cells. Consistent with decreased tumor burden, human sIL-6R level was significantly lower in CAR-T cells group (Supplementary Fig. 6B, C).

## Discussion

CAR-mediated tumor therapy has been associated with rapid and deep responses in a variety of hematologic malignancies, and in some of these patients, the responses appear to be maintained, even in heavily treated patients^3–6,23–26^. So far, CD19 and BCMA appear to be the most promising targets for B cell malignancies and MM, respectively. CAR-T cell therapy is expensive, in part, due to the restricted applicability of such targeted agents. Thus, a CAR-T construct that can be used to treat a wide variety of tumors may have broad appeal since this could result in a reduction in costs. Moreover, if a patient relapses after CAR-T cell therapy targeting those antigens, they may potentially be treated with CAR-T cells targeting a different antigen. We have shown that CD126 is aberrantly expressed in a wide variety of tumors—both hematologic and “solid” malignancies. The IL-6/Jak/Stat pathway is considered to be critical in many tumors by enabling cell growth and survival, promotes angiogenesis and metastases formation, and by creating a favorable immune microenvironment for the tumor^17^. While the role of CD126 itself in such tumors is not clear, we found that expression of the protein is associated with a worse prognosis. This fits well with reports that in a variety of tumors, high serum IL-6 levels correlate with a less favorable prognosis, including MM^27^, head and neck cancer^28^, non-small cell lung cancer^29^, colorectal carcinoma, and ovarian carcinoma^30^. Moreover, high IL-6 levels are often seen in other tumors, including breast cancer^31^, pancreatic^32^, and prostate adenocarcinoma^33^, as well as advanced renal cell carcinoma^34^. Current trials with tocilizumab, a monoclonal antibody that binds to CD126 are underway in chronic lymphocytic leukemia, breast, ovarian, and pancreatic cancer^17^, lend further support for our approach to target this antigen with CAR-T cells.

The vast majority of tumor cell lines that express CD126 were effectively killed in a dose-dependent manner in vitro using our CAR-T construct. Target cell killing was specifically dependent on the expression of this antigen and downregulation of CD126-abrogated CAR-T cell killing. We utilized two in vivo models that express CD126 for tumor control (MM and prostate adenocarcinoma), and in both scenarios, the CAR-T cells infiltrated the tumor xenografts, were activated, expanded, and led to tumor cytoreduction. Given that CD126 exists in a soluble form (sIL-6R) in human plasma^35^ and our construct binds to this protein, we evaluated whether the presence of sIL-6R will interfere with CAR-T cell killing. Fortunately, sIL-6R did not appear to abrogate CAR-T cell-mediated tumor cell killing neither in vitro nor in vivo. Indeed, even in vitro, many cell lines that express CD126 release large amounts of this protein in the media and this also did not interfere with CAR-T cell killing of target cells.

A critical organ where normal cells express CD126 is the liver. One of the potential toxicities of tocilizumab is transaminitis. Therefore, we evaluated whether our CAR-T cells bind to murine CD126 and whether the cells can induce hepatotoxicity. Fortunately, although the cells do bind mCD126, we did not observe any evidence of hepatotoxicity over a 2-week observation period in mice. There was no difference in murine behavior, weight loss, or evidence of hepatic dysfunction at the end of the observation period.

Given the ubiquitous expression of CD126 on hematopoietic cells, we also tested the potential toxicity of a CAR-T construct that targets such an antigen on a variety of immune cells. We found that CD126 targeting CAR-T cells did not have any impact on autologous PBMCs, B cells, T cells, monocytes, and NK cells.

One of the most common complications of CAR-T cell therapy is cytokine release syndrome that is mediated by IL-1 and IL-6^36,37^. This is supported by the high clinical efficacy of tocilizumab to treat this syndrome^36,38^. A potential benefit of our construct is that it may be associated with a lower risk of cytokine release syndrome (CRS) since the cells may reduce sIL-6R levels and inhibit IL-6 trans-signaling^17^. Interestingly, in our in vivo studies, we observed lower levels of SAA-3, the equivalent of human C-reactive protein and an important surrogate of CRS in human studies in mice treated with the CD126 targeting construct compared to controls. It has been suggested that IL-6-targeted CAR-T cells may be used to treat CRS^39^. It appears from our studies that CD126 targeting CAR-T cells may not only have broad antitumor activity but may be associated with a low risk of CRS, at least in the murine model. Inhibition of trans-signaling may also have the added benefit of negating IL-6 responses in tumor cells that do not express CD126.

In summary, CD126 is expressed by many types of tumors. CAR-T constructs targeting CD126 are able to kill a wide variety of tumor cell lines both in vitro and in vivo. Despite the expression of CD126 on liver cells, such CAR-T cells were not toxic to the liver. Targeting CD126 by CAR-T cells could reduce the incidence of cytokine release syndrome.

## Supplementary information

Supplementary Information
