# Supplementary material for: Preclinical development of CD126 CAR-T cells with broad antitumor activity
Source: Blood Cancer J. 2021 Jan 4;11(1):3. doi: 10.1038/s41408-020-00405-z (PMC7791061; doi:10.1038/s41408-020-00405-z)
Supplement: Supplementary file 1 — Supplementary Information [file 41408_2020_405_MOESM1_ESM.pdf]

Supplementary information

Supplementary Figure 1. CD126 is ubiquitously expressed on tumor cells and is a prognostic marker. (A) CD126 mRNA expression (RMA log2 signal intensity) was measured across 36 cancer type cell lines (n=917). (B) Median Fluorescence intensity (MFI) of CD126 expression in $CD138^{+}$ multiple myeloma (n=24) cells isolated from bone marrow compared to normal plasma cells (n=7). (C) MFI for CD126 expression in $CD19^{+}$ ALL cells (n=5) compared to $CD19^{+}$ normal B-cells (n=7). (D) mRNA expression of CD126 in AML (n=173) compared normal control cells (n=70). Mann-Whitney U test: *P<0.05.

Supplementary Figure 2. Development of a CD126 targeted CAR. Transduction efficiency of primary T cells was estimated with flow cytometry using (A) a biotinylated recombinant human CD126 protein for scFv rhMP-1 and (B) mCherry reporter gene for scFv VQ8F11. (C) CD126 knock-down in RPMI cell line was confirmed with flow cytometry. (D) CD126 over-expression in K562 cell line was confirmed with flow cytometry. Comparison of the killing efficiency for two CD126 directed CAR, CD19 directed CAR and Mock T-cells were tested against (E) RPMI 8226, (F) U266 and (G) MM.1S cells. Bioluminescence cell killing was performed in ultrachilli-luciferase expressing cancer cell lines when co-cultured with either CAR-T or mock T cell at effector to target ratios of 10, 5, 1, 0.2 and 0.1 (n=12). Percent specific lysis was quantified after 16 hours of incubation. Two-way ANOVA and multiple comparison test. ns = not significant, *P<0.05, **P<0.01, ***P<0.001 and ****P<0.0001.

Supplementary Figure 3. CD126 targeted CAR-T cells demonstrated potent in vitro activity against multiple tumor cell lines. Bioluminescence cell killing was performed in ultrachilli-luciferase expressing cancer cell lines, (A) U266B1, (B) MM.1S, (C) KMS-12-PE, (D) SU-DHL-1, (E) KARPASS 299, (F) 624-mel, (G) Jurkat, (H) MOLT-4, (I) LNCaP, (J) Hep G2, (K) A549 and (L) U-251 MG when co-cultured with either CAR-T or mock T cell at effector to target ratios of 10, 5, 1, 0.2 and 0.1 (n=12). Percent specific lysis was quantified after 16 hours of incubation. Two-way ANOVA and multiple comparison test. ns = not significant, *P<0.05, **P<0.01, ***P<0.001 and ****P<0.0001.

Supplementary Figure 4. CD126 targeted CAR-T cells demonstrated potent in vitro activity against primary ALL cells. Cell killing of primary ALL cells from four patients was measured by calcein AM labelled cells when co-cultured with either CAR-T or mock T cells at effector to target ratio of 10, 5, 2.5, 1, 0.2 and 0.1 after 16 hours of incubation (n=10). Two-way ANOVA and multiple comparison test. Ns = non-significant, **P*<0.05, ***P*<0.01, ****P*<0.001 and *****P*<0.0001.

Supplementary Figure 5. CD126 targeted CAR-T cells bind to sIL6R and do not induce toxicity in a murine model. (A) sIL6R was measured in conditioned media from RPMI, HL-60, DU 145 and K562 cells by ELISA. Mock T cell or CAR-T cells were cultured in conditioned media from sIL6R producing cells for 12 hours and sIL6R was quantified in media supernatant with ELISA (n=3). (B) Bioluminescence cell killing was performed in ultrachilli-luciferase expressing RPMI when co-cultured with CAR-T cells at effector to target ratios of 10, 5, 2.5 and 1 (n=12) with or without recombinant human sIL6R (50ng/ml). Percentage specific lysis was quantified after 16 hours of incubation. (C) Binding affinity for mouse IL6R was determined by flow cytometry after incubating with increasing dose of mouse IL6R-His and anti-His-PE antibody. (D) Serial body weight measurements for mice that either received mock T cells (n=5) or CAR-T cells (n=5). (E) Weight of the explanted liver in mice, 2 weeks after injection with mock T cells (n=5) or CAR-T cells (n=5). (F) Aspartate aminotransferase (AST) was measured in plasma of mice injected with mock T cells (n=5) or CAR-T cells (n=4). (G) Mouse SAA-3 was measured in murine plasma 2 weeks after injection with either mock T cells (n=5) or CAR-T cells (n=4). (H) Human IFNγ was measured in murine plasma 2 weeks after injection with mock T cells (n=5) or CAR-T cells (n=4). Mann-Whitney *U* test. Two-way ANOVA and multiple comparison test. NS = not significant, **P*<0.05, ***P*<0.01, ****P*<0.001 and *****P*<0.0001.

Supplementary Figure 6. CD126 targeted CAR-T cells have potent antitumor activity but do not kill autologous immune cells. (A) Tumor burden (D-luciferin BLI of DU-145-ultrachilli-luciferase) mice were quantified on day 10, 14 and 16. (B) ELISA was performed to quantify plasma level of human IFNγ, mouse SAA-3 and human s-IL6R in mice with RPMI-ultrachilli-luciferase expressing tumor xenografts that either

received mock T cells (n=5) or CAR-T cells (n=5). (C) ELISA was performed to quantify plasma level of human IFNγ, mouse SAA-3 and human s-IL6R in mice (DU145-ultrachilli-luciferase) that either received mock T cells (n=5) or CAR-T cells (n=5). CD126 targeted CAR-T cells do not kill autologous PBMC or B cells, T cells, monocytes, or NK cells. (D) RPMI 8226 cells were used as a positive control for the assay. RPMI 8226 killing was measured by flow cytometry. (E) PBMC killing was measured by flow cytometry by quantifying CFSE labelled cells when co-cultured with mock T-cells or CD126 targeted CAR-T cells. B cell killing was determined with flow cytometry by quantifying CD19 positive cells in CFSE labelled PBMC cells when co-cultured with mock T-cells or CD126 targeted CAR-T cells. T-cell killing was determined by flow cytometry by quantifying the fraction of CD3 positive cells in CFSE labelled PBMC cells after co-culture with mock T-cells or CD126 targeted CAR-T cells. Monocyte killing was measured by flow cytometry by quantifying the fraction of CD14 positive cells in CFSE labelled PBMC cells when co-cultured with mock T-cells or CD126 targeted CAR-T cells. NK cell killing was measured by flow cytometry by quantifying the fraction of CD56 positive cells in CFSE labelled PBMC cells when co-cultured with mock T-cells or CD126 targeted CAR-T cells. n=3, healthy human donor. Mann-Whitney *U* non-parametric test. Two-way ANOVA and multiple comparison test. ns = not significant, *$P<0.05$, **$P<0.01$, ***$P<0.001$ and ****$P<0.0001$.

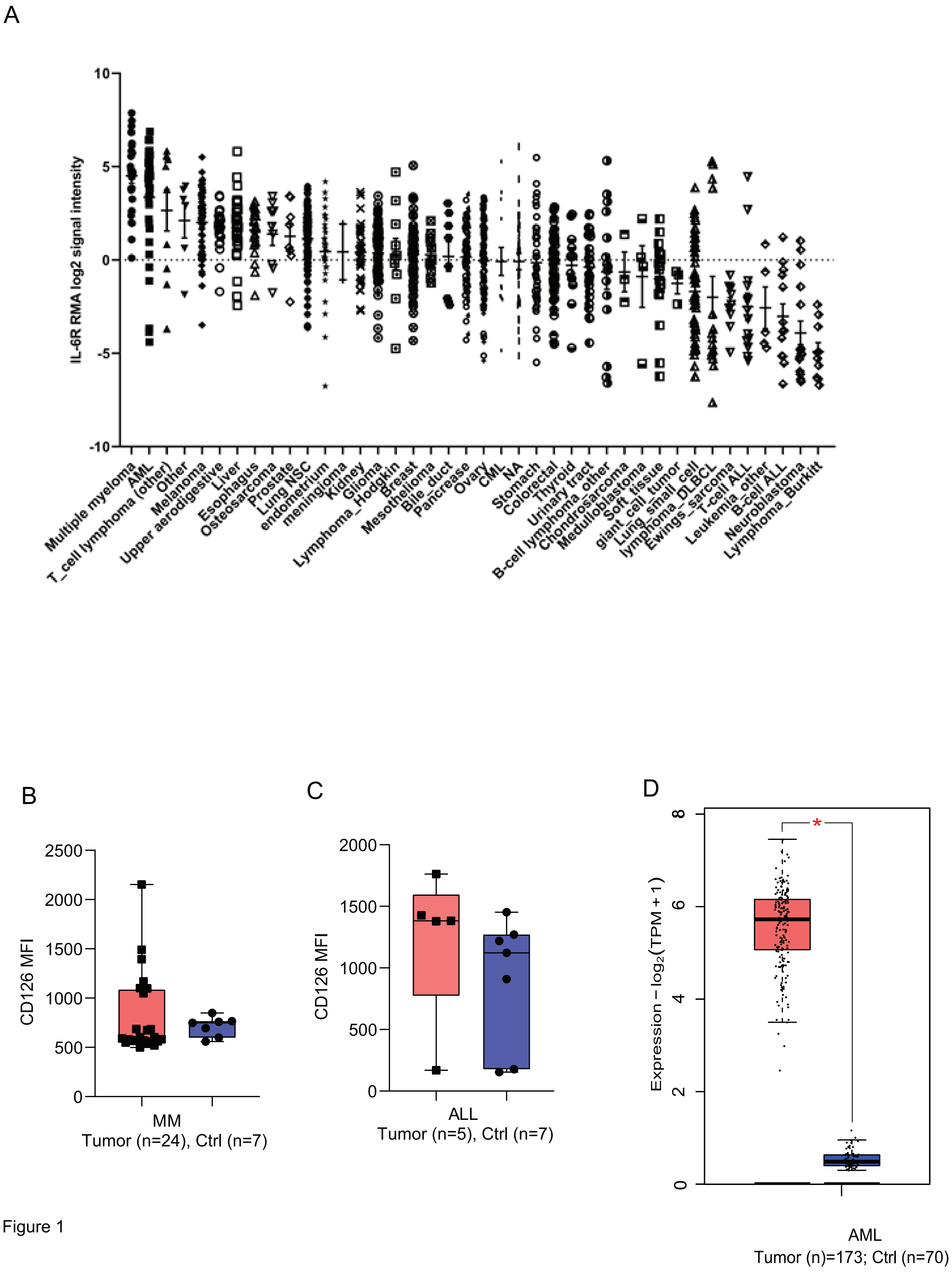
A
10
5
0
-5
-10
IL-6R RMA log2 signal intensity
Multiple myeloma
AML
T_cell lymphoma (other)
Other
Melanoma
Upper aerodigestive
Liver
Esophagus
Osteosarcoma
Prostate
Lung NSC
endometrium
meningioma
Kidney
Glioma
Lymphoma_Hodgkin
Breast
Mesothelioma
Bile_duct
Pancrease
Ovary
CML
NA
Stomach
Colorectal
Thyroid
Urinary tract
B-cell lymphoma_other
Chondrosarcoma
Medulloblastoma
Soft_tissue
giant_cell_tumor
Lung_small_cell
lymphoma_DLBCL
Ewings_sarcoma
T-cell ALL
Leukemia_other
B-cell ALL
Neuroblastoma
Lymphoma_Burkitt
B
2500
2000
1500
1000
500
0
CD126 MFI
MM
Tumor (n=24), Ctrl (n=7)
C
2000
1500
1000
500
0
CD126 MFI
ALL
Tumor (n=5), Ctrl (n=7)
D
8
6
4
2
0
Expression – log2(TPM + 1)
*
AML
Tumor (n)=173; Ctrl (n=70)

Figure 1

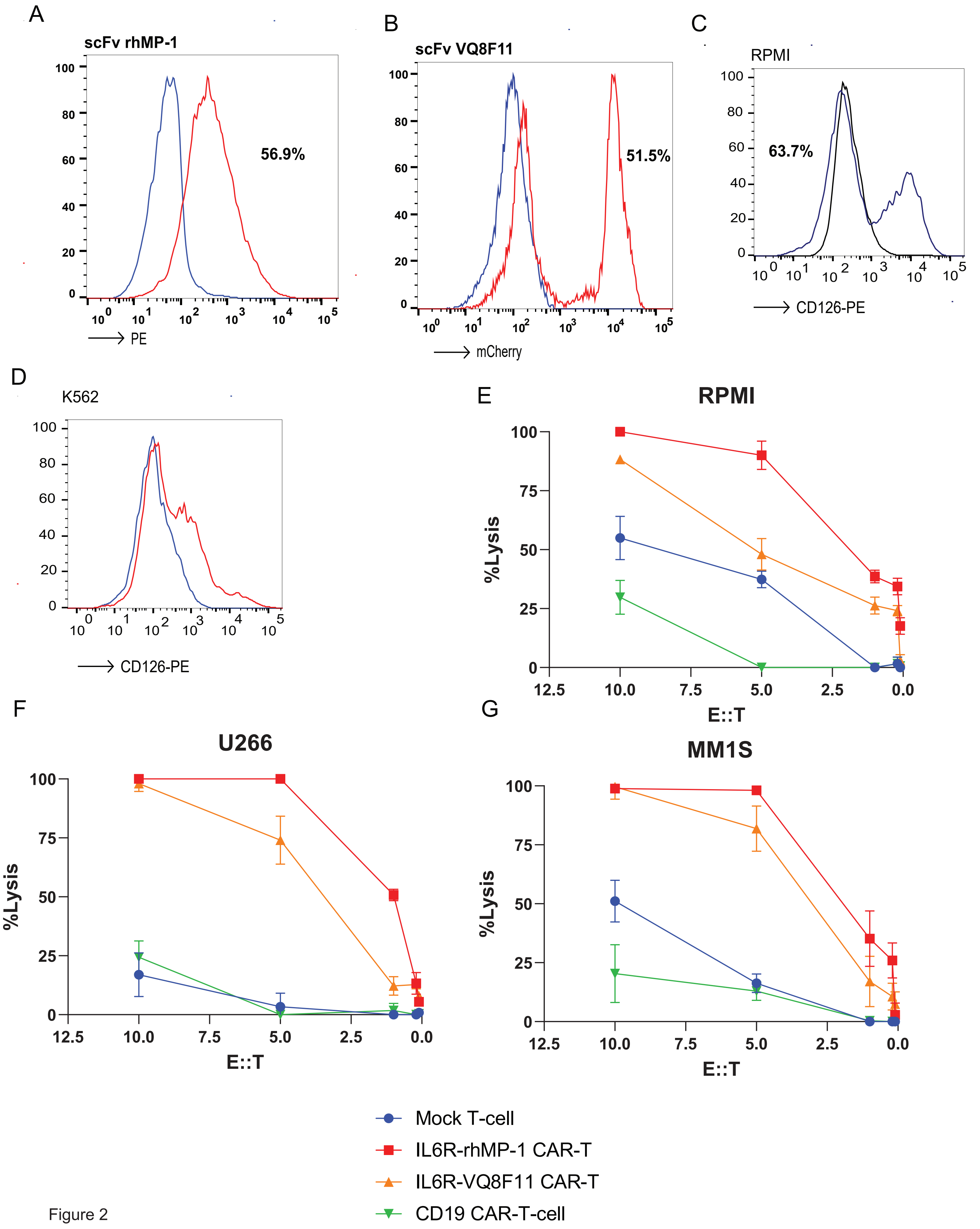
A
scFv rhMP-1
56.9%
PE
B
scFv VQ8F11
51.5%
mCherry
C
RPMI
63.7%
CD126-PE
D
K562
CD126-PE
E
RPMI
%Lysis
E::T
F
U266
%Lysis
E::T
G
MM1S
%Lysis
E::T
Mock T-cell
IL6R-rhMP-1 CAR-T
IL6R-VQ8F11 CAR-T
CD19 CAR-T-cell

Figure 2

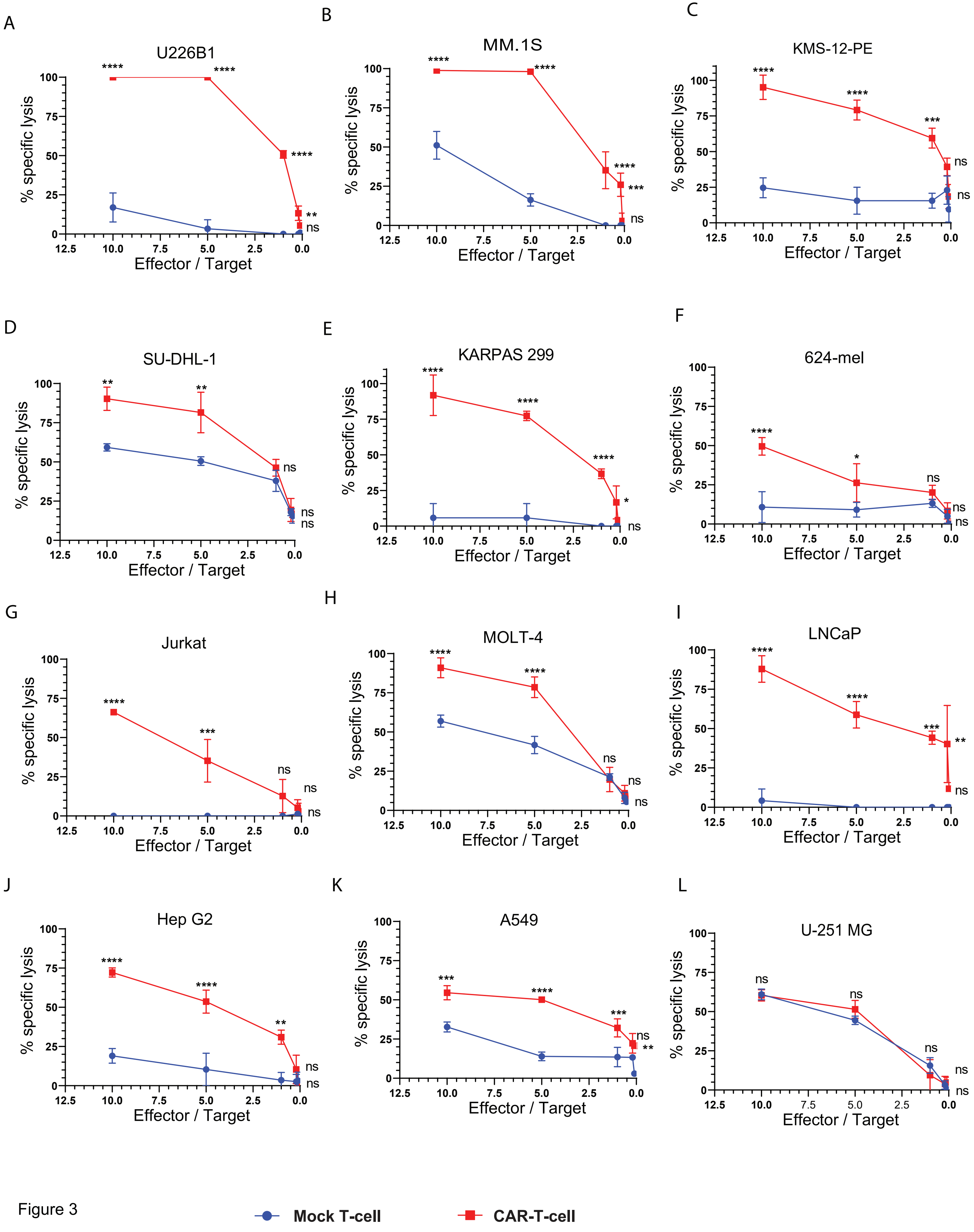

A
U226B1
B
MM.1S
C
KMS-12-PE
D
SU-DHL-1
E
KARPAS 299
F
624-mel
G
Jurkat
H
MOLT-4
I
LNCaP
J
Hep G2
K
A549
L
U-251 MG
% specific lysis
Effector / Target
Mock T-cell
CAR-T-cell

Figure 3

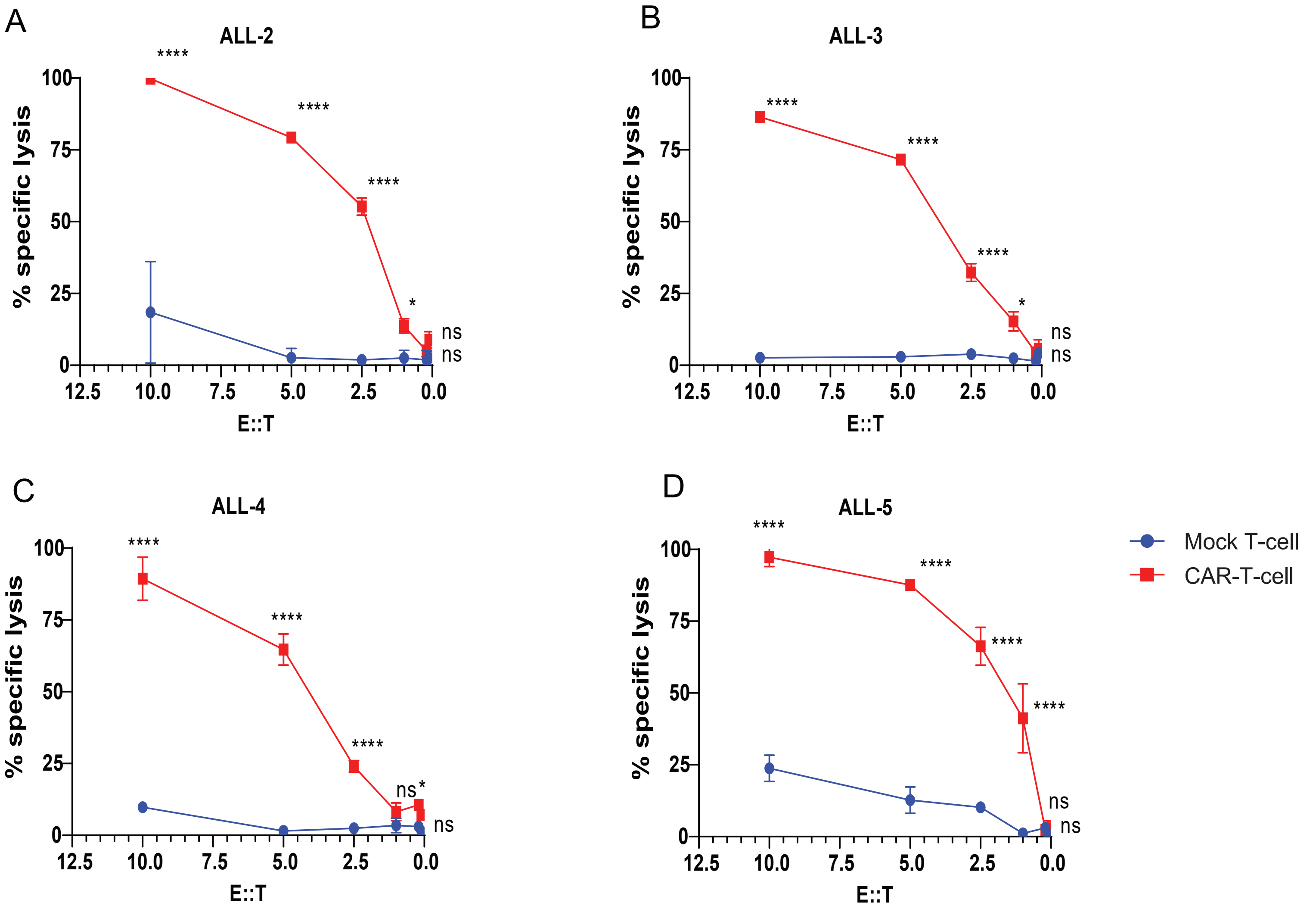
A
ALL-2
B
ALL-3
C
ALL-4
D
ALL-5
% specific lysis
E::T
****
*
ns
ns*
Mock T-cell
CAR-T-cell

Figure 4

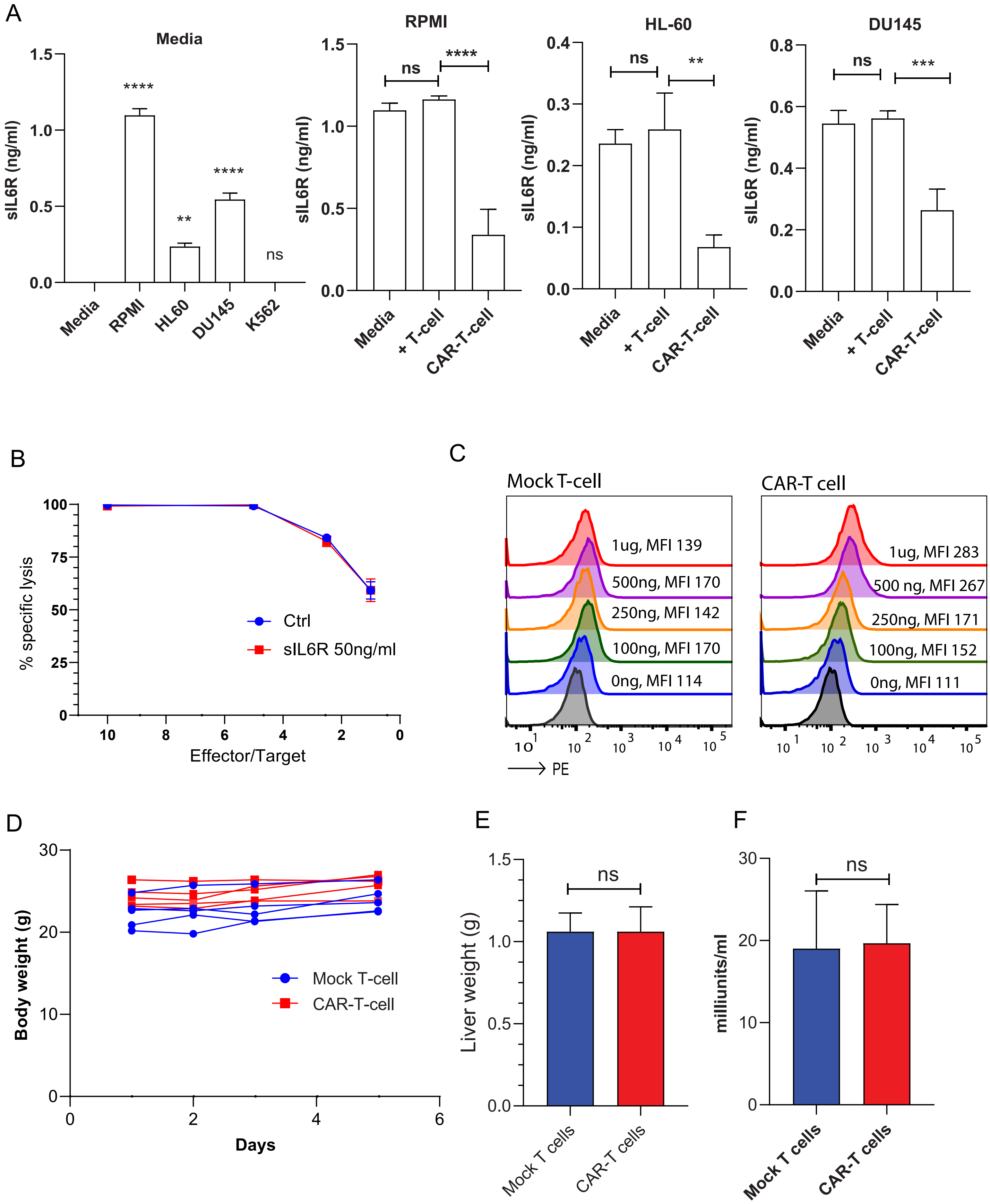
A
Media
sIL6R (ng/ml)
****
**
****
ns
Media
RPMI
HL60
DU145
K562
RPMI
HL-60
DU145
ns
****
***
+ T-cell
CAR-T-cell
B
% specific lysis
Ctrl
sIL6R 50ng/ml
Effector/Target
C
Mock T-cell
CAR-T cell
1ug, MFI 139
500ng, MFI 170
250ng, MFI 142
100ng, MFI 170
0ng, MFI 114
1ug, MFI 283
500 ng, MFI 267
250ng, MFI 171
100ng, MFI 152
0ng, MFI 111
PE
D
Body weight (g)
Mock T-cell
CAR-T-cell
Days
E
Liver weight (g)
Mock T cells
CAR-T cells
F
milliunits/ml

Figure 5

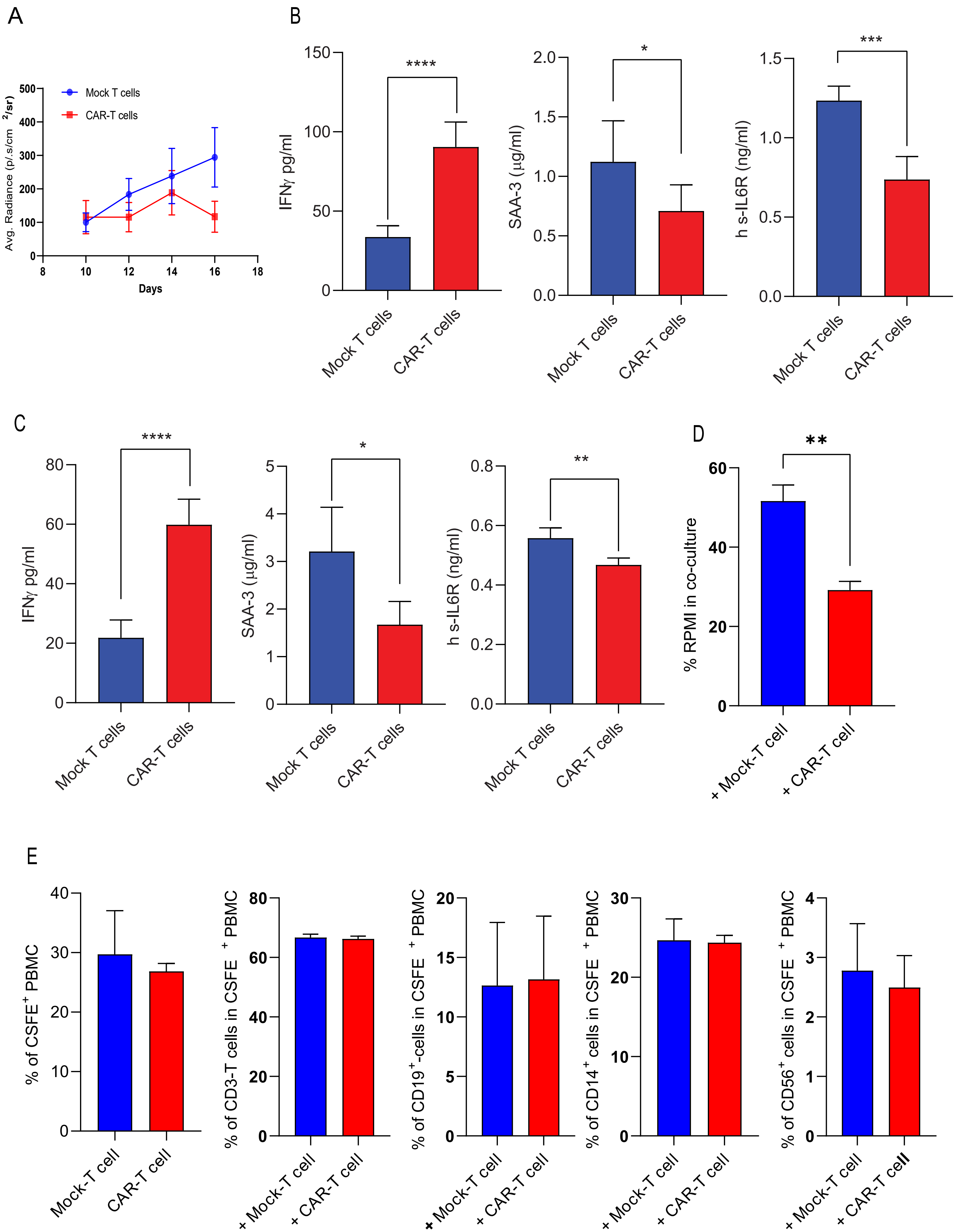

A
B
C
D
E
Avg. Radiance (p/.s/cm ²/sr)
Mock T cells
CAR-T cells
Days
IFNγ pg/ml
SAA-3 (µg/ml)
h s-IL6R (ng/ml)
% RPMI in co-culture
% of CSFE⁺ PBMC
% of CD3-T cells in CSFE ⁺ PBMC
% of CD19⁺-cells in CSFE ⁺ PBMC
% of CD14⁺cells in CSFE ⁺ PBMC
% of CD56⁺ cells in CSFE ⁺ PBMC
+ Mock-T cell
+ CAR-T cell
Mock-T cell
CAR-T cell

Figure 6
